# A Case of Primary Non-Hodgkin's Lymphoma of the External Auditory Canal

**DOI:** 10.1155/2013/138397

**Published:** 2013-08-04

**Authors:** Luca Bruschini, Andrea De Vito, Susanna Fortunato, Marco Pelosini, Giulia Cervetti, Mario Petrini, Stefano Berrettini

**Affiliations:** ^1^Head-Neck Department, ENT Audiology and Phoniatry Unit, University Hospital of Pisa, Via Paradisa 2, 56100 Pisa, Italy; ^2^Hematology Unit, University Hospital of Pisa, Via Paradisa 2, 56100 Pisa, Italy

## Abstract

Lymphomas represent the second most frequent malignant tumor (incidence 2.5%) in the head and neck region. Non-Hodgkin lymphomas (NHLs) present with cervical lymph node involvement, but in 40% extranodal site could be primary involved: nasopharynx, the lacrimal sac, the temporal bone, or the others areas. NHLs of the ear are rarely reported. In this report, we described a patient with primary NHL of the external ear canal who was successfully treated with surgical excision and chemotherapy.

## 1. Introduction 

Lymphomas represent the second most frequent malignant tumor (incidence 2.5%) in the head and neck region [1, 2]. Non Hodgkin lymphomas (NHLs) present with cervical lymph node involvement, but in 40% extranodal site could be primary involved: nasopharynx, the lacrimal sac, the temporal bone, or the other areas [3].

NHL of the ear are rarely reported. Ogawa et al. found only eighteen reported cases of primary temporal bone lymphoma in the English and Japanese literature, including the cases of primary lymphoma of the middle ear [4]. These tumors of the temporal bone are typically associated with facial nerve paralysis and hearing loss, and few cases have a soft mass in the external auditory canal too [5, 6].

Even more rarely, primary NHLs arise from the external auditory canal (EAC) and fill it [7, 8]. Only eight cases of lymphoreticular tumors of the EAC are described in the international literature [2, 7, 9–12]. Thus, the histological and clinical features of primary EAC lymphoma have not been extensively characterized.

In this report, we described patient with primary NHL of the EAC who was successfully treated with surgical excision and chemotherapy. A review of the literature regarding EAC lymphoma also is provided to describe the characteristics and management options for this uncommon manifestation of the NHL. 

## 2. Case Report

**Figure 1:**
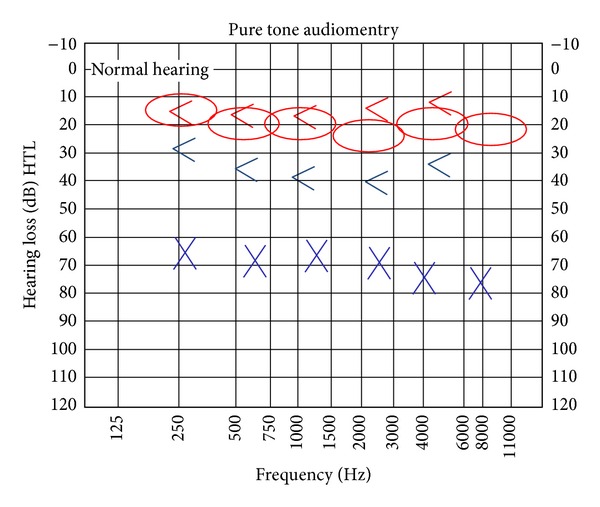
Left conductive hearing loss.

D.G.E., a 46-year-old man, came to our attention in March 2009 for a left hearing loss started 3 weeks before. The patient referred only to aural fullness and he did not report any other symptoms like earache or effusion. Otomicroscopy revealed a polypus occluding the left EAC. The tympanic membrane was not visible. Pure tone audiometry showed a left conductive hearing loss and a normal hearing threshold at right (Figure 1). This peculiar clinical picture could be referred to a middle ear otitis with granulation tissue in external ear, but the absence of aural fullness, othorrea was atypical for this diagnosis.

**Figure 2:**
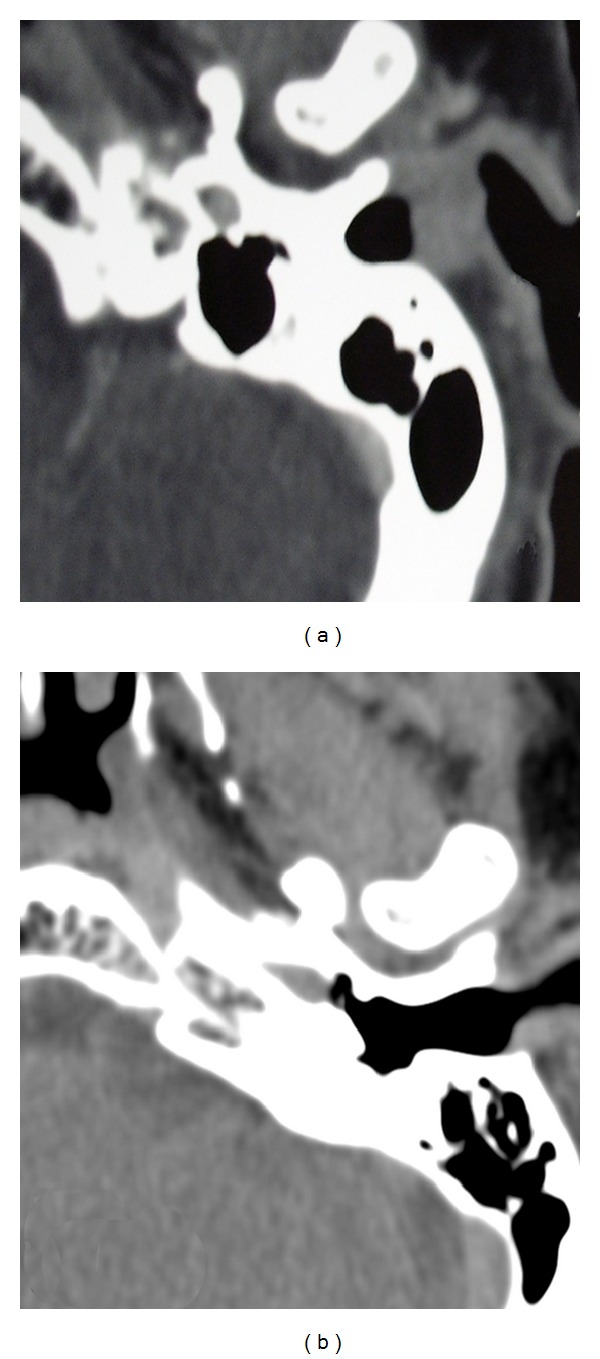
Petrous bone CT before (a) and after (b) the excisional biopsy.

Therefore, a petrous bone computed tomography (CT) was performed. The CT scan showed a pedunculated polypoid mass occluding only the lateral portion of the EAC, with a normally aerated middle ear and mastoid, and a normal bone of the EAC without signs of erosion (Figure 2(a)).

We carried out a excisional biopsy under local anesthesia. The histologic examination of the mass revealed a peripheral B-cell NHL: diffuse large cells 60% and follicular 40%, with an immune-proliferative activity (Ki-67 index) of 60%. Immunoperoxidase staining of the atypical cells was positive for CD20, bcl2, bcl6, CD10, CD21 and negative for CD3, CD5, CiclineD1, and CD30. Clinical staging was completed with a total-body Positron Emission Tomography (PET), a CT scan of the chest and abdomen, and a percutaneous bone marrow biopsy were performed which resulted negative for other lymphoma's localitation. 

The patient was admitted to the Hematology Clinic and treated with six cycles of immunopolychemotherapy according to R-CHOP scheme (Cyclophosphamide, Adriablastin, Vincristine, Rituximab, and methylprednisolone). 

At 24 months, follow-up evaluation confirms stable complete remission. No localized recurrence or any systemic involvement was detected. An ear examination did not reveal any anomaly. The tympanic membrane was normal; the hearing threshold as well as the EAC was normal. A CT scan, performed after 9 months of follow-up showed a normal EAC (Figure 2(b)).

## 3. Discussion 

Although lymphomas are frequently observed in the head and neck compartment, they rarely involve EAC [7]. To our knowledge only eight cases of primary lymphoma (six patients) of the EAC are reported in the literature with diagnosis of B-cell lymphoma in seven cases (two patients had bilateral lymphoma of the EAC), one T-cell lymphoma, and one anaplastic lymphoma. 

The clinical presentation of primary lymphomas of the EAC is highly aspecific. Therefore these malignant disorders are usually misdiagnosed and treated as external otitis. 

As the lesion grows there is a sensation of blocked ear, transmission hearing loss, and increasing earache, sometimes without otorrhea or otorrhagia. Diagnosis is done by histological studies [7].

In a recent review, Delgado et al. reported a 53-year-old woman with a Non Hodgkin's T cell lymphoma of the EAC [7]. The patient referred only to nonspecific discomfort in the ear and gradual hearing loss over a period of one year. Merkus et al. described an 83-year-old woman with an anaplastic large cell lymphoma of the EAC skin [2]. Another old woman, 83-year-old, had a B-cell lymphoma [9]. This patient had fullness and hearing loss. Shuto et al. described a 49-year-old man with a B-cell lymphoma of both EACs [10]. Also Maiche et al. reported one patient affected by a bilateral lymphoma of the EACs [12]. This patient had a history of being a hearing aids user for a long period of time [12]. Fish et al. described a 53-year-old female with a B-cell lymphoma of the EAC [11]. Unlike the others, this patient presented with pain in her ear radiating to her jaw. Besides, she had been treated for otitis externa. A total of seven patients and nine lymphomas, including the case herein reported, are summarized in Table 1.

Sometimes a single biopsy could not be sufficient, as described by Delgado et al. [7]. An incisional biopsy may cause a misdiagnosis or a delay in diagnosis, as reported in the literature [11].

In our case we decided for surgical excision of the whole lesion, considering also CT scans which showed absence of bone lesions, that allowed right and early diagnosis of NHL of EAC.

The aspecific clinical picture makes diagnosis not easy: differential diagnosis includes benign, malignant process of the EAC, and external and media otitis. The lack of earache and of bone erosion (cleared with CT scan) made us rule out the malignant otitis external. 

 International guidelines for therapy have not been established yet due to the scanty number of cases.

The surgical treatment of the EAC tumors of epithelial origin is largely accepted. However, in cases of high grade lymphomas or disseminated disease radiotherapy and/or chemotherapy could be considered as the primary and definitive treatments. For an isolate lymphomas tumor of the EAC, the surgical procedure should consist only in the excision biopsy of the tissue mass. 

This case was treated with excisional biopsy and six cycles of immunopolychemotherapy, according to known literature. Delgado et al. reported similar procedures, excisional biopsy and chemotherapy for one patient [7], as well as for the patient reported by Shuto et al. [10]. In the last mentioned case, the tumor did not respond to polychemotherapy; thus, the surgical resection was evaluated as the best treatment [10]. Only one patient underwent an aggressive procedure with a lateral temporal bone resection after three cycles of chemotherapy [11]. The external beam radiation was the therapy chosen for another patient reported in the literature [9]. And it could be considered an alternative treatment for single disease like the reported case.

The reported case highlights that malignant lymphoma should be suspected in case of a tissue mass of the EAC especially if not associated to ear discharge and not responsive to conventional treatment.

Lymphoma arising from the EAC appears to be a particular biological entity and is commonly associated with a good prognosis. Novel strategies in the improvement of prognosis should be addressed in future studies by assessing prognostic factors implicated. Our case suggests that an excisional biopsy may be the best way for an early and right diagnosis and confirms that surgical treatment followed by chemotherapy could be an effective treatment.

## Figures and Tables

**Table 1:** Age, sex, phenotype, symptoms, and management of seven patients and nine lymphomas. All the cases are described in the scientific literature.

Case number	Age (years)	Sex	Ear	Histology	Immune phenotype	Symptoms	Biopsy	Therapy	Outcome	Reference
1	53	F	Left	Anaplastic	T cell	Hearing loss	Excisional	Chemotherapy	Alive	[7]
2	83	F		Anaplastic		Hearing loss	Incisional	Radiotherapy	Alive	[2]
3	83	F			B cell	Hearing loss	Incisional	Radiotharapy	Alive	[9]
4	49	M	Both		B cell	Hearing loss	Incisional	Surgery + chemotherapy	Alive	[10]
5		M	Both		B cell	Hearing loss	Incisional	Chemotherapy	Alive	[12]
6	53	F			B cell	Pain	Incisional	Surgery + chemotherapy	Alive	[11]
7	46	M	Left		B cell	Hearing loss	Excisional	Chemotherapy	Alive	Present study
